# Editorial: Women in food chemistry

**DOI:** 10.3389/fnut.2023.1227558

**Published:** 2023-07-04

**Authors:** Yasmina Sultanbawa, Dharini Sivakumar, Michele Iskandar

**Affiliations:** ^1^Centre for Nutrition and Food Sciences ARC Training Centre for Uniquely Australian Foods, Queensland Alliance for Agriculture and Food Innovation (QAAFI), The University of Queensland, Brisbane, QLD, Australia; ^2^Phytochemical Food Network Research Group, Department of Crop Sciences, Tshwane University of Technology, Pretoria, South Africa; ^3^School of Human Nutrition, McGill University, Macdonald Campus, Sainte-Anne-de-Bellevue, QC, Canada

**Keywords:** gender-equity, phytonutrients, plant foods, STEM fields, nutrition

In this Research Topic of Frontiers in Nutrition, we are pleased to present the introductory collection of articles on “*Women in food chemistry*.” The importance of science and gender equality is emphasized by UNESCO. To change cultural norms, a gender-equal society must be fostered, stigmas eliminated, and STEM careers should be encouraged for women and girls. This platform is therefore an excellent way to promote the work of women scientists across all fields of nutrition at Frontiers in Nutrition. The research presented here illustrates the diversity of research performed across the spectrum of Nutrition and Food Chemistry. This Research Topic included one review article and three research articles. The first author or last authors of the publications are women researchers.

Shoko et al. investigated the functional compounds, volatiles, and antioxidant properties of coriander leaves (*Coriandrum sativum*) stored under different light conditions during postharvest storage. The use of LED light as a post-harvest treatment improved the antioxidant properties and retained the acceptable aroma profiles of coriander leaves. Further, cold storage of coriander leaves in PET punnets exposed to blue LED light extended its storage life to 9 days. These findings indicate that this technology could be applied to supermarket storage and retail display shelves.

Additionally, Phan et al. investigated Kakadu plum's (*Terminalia ferdinandiana* Exell) (Kakadu plum) physicochemical properties, hydrolysable tannins, and antioxidant capacity. The Kakadu plum is a native Australian fruit that is wild harvested, and little is known about how maturity influences its phytonutritional properties and bioactivity. The total soluble solids, titratable acidity, and sugars increased during the fruit development from highly immature (< 25% degree of fruit fullness) to fully mature stages (75–100% degree of fruit fullness), whereas phenolic compounds, including major hydrolysable tannins, decreased with fruit ripeness. Such a study can assist indigenous enterprises, the food industry and other industries in selecting the best maturation stages for using Kakadu plum as a functional food ingredient.

An investigation of the fortification and bioavailability of saffron apocarotenoids in potato tubers was carried out by Gómez et al.. As a consequence of their metabolic, physiological, and ecological properties, apocarotenoids are becoming increasingly popular. In response to the enzymatic or reactive oxygen species-induced cleavage of carotenoids, apocarotenoids are produced. Two saffron apocarotenoids, crocins and picrocrocin, are high-value pigments used in food, feed, and pharmaceuticals. Crocins and picrocrocin are produced in potato tubers through the expression of a unique construct of genes responsible for the biosynthesis of saffron apocarotenoids. Crocins and picrocrocin have high bioaccessibility, and potatoes can accumulate them, thereby providing consumers with a greater range of health benefits.

Granado-Rodríguez et al. reported on quinoa seeds and the nutritional and quality changes that occur under different storage conditions. This article is important in the context of climate change as it has an impact on global food security in reference to long-term food availability and next-season seed preservation. To retain the nutritional quality, it is recommended to store the seed at low storage temperatures (4°C) and use hermetic bags to maintain the seed moisture content lower than 12%, which will increase the economic value as well as the seed viability.

## Author contributions

All authors listed have made a substantial, direct, and intellectual contribution to the work and approved it for publication.

